# Upgrading the Performance of Cholesteric Liquid Crystal Lasers: Improvement Margins and Limitations

**DOI:** 10.3390/ma11010005

**Published:** 2017-12-21

**Authors:** Josu Ortega, César L. Folcia, Jesús Etxebarria

**Affiliations:** 1 Department of Applied Physics II, Faculty of Science and Technology, University of the Basque Country, UPV/EHU, 48080 Bilbao, Spain; josu.ortega@ehu.eus; 2 Department of Condensed Matter Physics, Faculty of Science and Technology, University of the Basque Country, UPV/EHU, 48080 Bilbao, Spain; cesar.folcia@ehu.eus

**Keywords:** cholesteric liquid crystals, lasers, photonic materials

## Abstract

The topic of cholesteric-liquid-crystal lasers is a rapidly expanding research area in the field of soft-matter photonics. The increasing interest in this field is due to the high versatility that these lasers may possibly present and the prospects of giving rise to new miniaturized devices. However, further improvements in their operation capabilities are still required for potential applications. In this paper, we critically analyze the main strategies proposed up to now to optimize their performance. We show theoretically and experimentally that possible innovations in the device structure cannot produce lasers with threshold energies below a certain limit. This limit is determined by the light scattering and absorption losses inside the liquid crystal. Even assuming the case of samples free of defects and perfectly non-absorbing, an intrinsic light scattering, typical of mesogens, still remains. Numerical estimates of the thresholds indicate that these lasers could hardly be driven by compact light sources such as current electroluminescent or light-emitting diodes. Since the improvement possibilities regarding cell architecture seem to be exhausted, the advance must come from the use of new dye molecules. These molecules should show enhanced emission cross-sections and be efficiently integrable within the mesogenic solvent. In addition, the fluorescent systems must present very small quantum yields to triplet states if continuous-wave lasing is sought. In this respect, quantum dots are an alternative to explore for further investigations.

## 1. Introduction

In cholesteric liquid crystals (CLC), molecules are self-assembled forming a helical structure. When the pitch of the CLC helix is of the order of magnitude of visible light wavelength, the material can exhibit photonic properties. In these cases CLCs present a photonic band gap (PBG), and circularly polarized light with the same handedness as that of the helix cannot propagate in a certain range of frequencies. The photonic character of CLCs is the basis for the application of these materials as low-threshold distributed feedback (DFB) lasers. In fact, when CLCs are doped with fluorescent dyes, mirrorless laser generation can be obtained at the edges of the PBG. Since the discovery of laser emission in CLCs by Kopp et al. in 1998 [1], many efforts have been made to build up CLC lasers with increasing performance and durability [2,3,4,5,6,7,8,9,10,11,12,13,14,15,16]. 

Apart from liquid crystal (LC) lasers based on the self-assembled helical structure, there are other DFB laser types grounded on artificially designed structures such as gratings [17,18,19,20]. On the other hand, CLCs have also been used in connection with other laser mechanisms such a leaky mode lasers [3] and random lasers [21,22,23], and references therein. However, in the present work we are going to focus ourselves exclusively on the so-called edge-mode and defect-mode CLC lasers. 

The main features that make CLC lasers so attractive are the ease of fabrication that permits to obtain miniaturized devices, the low threshold intensity, and the possibility of tuning the wavelength by using different emission angles [24], pitch gradients [25,26], or external stimuli such as electric field application [27,28,29,30], light irradiation [31,32,33], mechanical stretching [34] or temperature [35,36]. 

However, in order to manufacture small laser devices economically and at great scale it is indispensable to further improve their performance. One of the key points is reducing the threshold of the pumping energy required for laser emission, since it would permit using simpler pumping sources. Up to now, pumping sources have always been bulky high-power pulsed lasers with pulse duration in the range of nano- to pico-seconds. 

**Figure 1:**
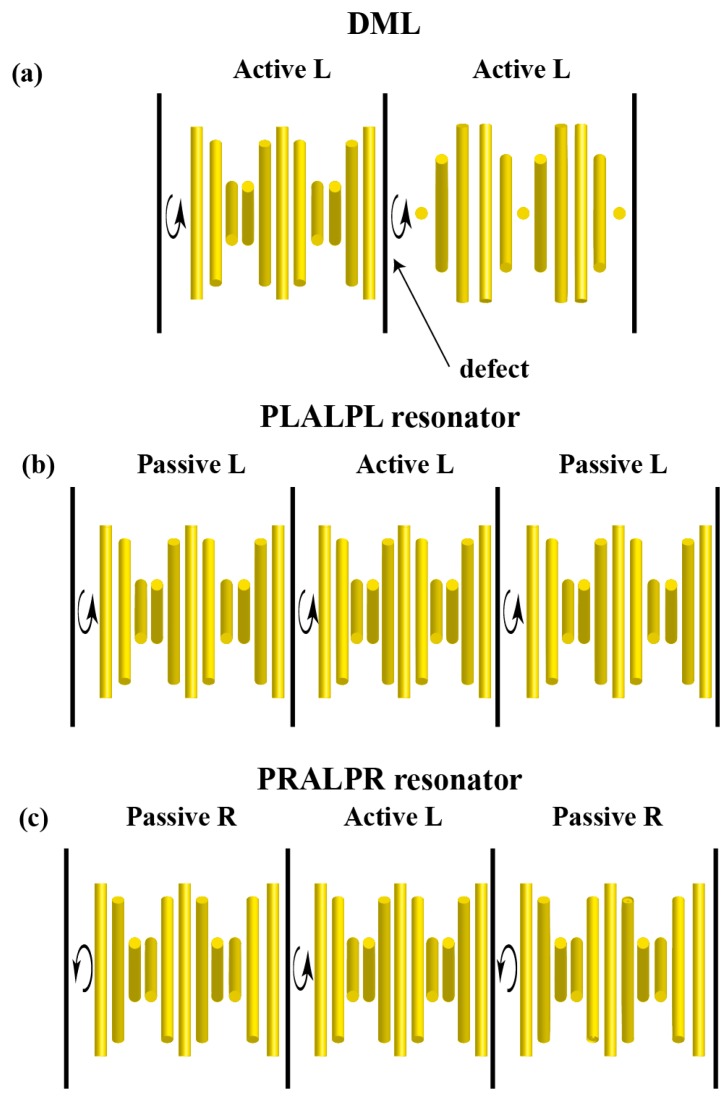
Schematic representation of different cell architectures. (**a**) Bilayer CLC (cholesteric liquid crystals) cell with a defect consisting in a jump of π/2 in the twisting angle; (**b**,**c**) are complex CLC structures with a dye-doped layer sandwiched between two passive layers acting as mirrors. In (**b**) the whole structure presents the same handedness, i.e., L—left, whereas in (**c**), the external films have opposite handedness, i.e., R—right, to that of the active film. The setups are labeled as DML (defect mode laser) (**a**), PLALPL (passive left handed layer-active left handed layer-passive left handed layer) resonator (**b**), and PRALPR (passive right handed layer-active left handed layer-passive right handed layer) resonator (**c**).

One of the most effective strategies to reduce the pumping threshold energy is the use of multilayer CLC cells. Some of these architectures are based on the so-called defect mode laser (DML). The possibility of lasing originated by defects was theoretically predicted in several works [37,38,39,40,41]. Although the concept of DML is used in a broad sense in the literature, hereafter we will refer to it as the laser mode that appears when a dye-doped CLC layer presents a structural distortion. We will pay special attention to the case of a phase jump in the twisting angle of the LC director as we move along the helix axis (see Figure 1a). The DML presents in general a lower threshold than that of the edge mode (EM) of the undistorted structure. In this respect Schmidtke et al. [42] reported a reduction of about one order of magnitude in the laser threshold by stacking two layers of cholesteric polymer films with a phase jump of π/2. A decrease of about one fourth of the threshold energy was also reported by Jeong et al. [43] in a cell formed by two dye-doped polymeric-CLC films with a thin rubbed defect layer in between. Other studies concerning irregularities in the helical pitch have been also carried out and, also in these cases, the DML has shown a lower threshold than that of the corresponding EM [44].

An alternative to the DML approach is the use of other types of multilayer cells, usually three layers, in which the two external films act as reflectors. The laser modes that appear in these cell architectures are also considered in the literature as defect modes. However, in most of the cases, the external films are inactive and only the internal layer is dye-doped. Therefore, the role of the reflectors is similar to that of the mirrors in Fabry-Perot cavity lasers. This strategy has been used in many different ways: In some cases two polymeric CLC passive structures sandwiching a dye-doped nematic or isotropic layer [45], in other cases, an active polymeric CLC film placed between a conventional mirror and a CLC passive structure [46]. Other possibilities include the case of an active CLC layer inserted between two CLC passive layers of the same handedness (Figure 1b) [47], or of the opposite to the one of the active film (Figure 1c) [48]. In all previous cases, polymeric CLC’s were used to achieve a good optical contact between the different layers. In order to avoid the laborious construction of polymeric structures and further simplify the construction of complex cells, Zhou et al. [49] stacked different low-molecular CLC mixtures by using standard glass cells. In this case, a remarkable improvement in the quality of the laser cavity was also found. 

The previous studies present, in general, important improvements in the quality of the laser cavities. However, in some cases the threshold energies were compared to simple active CLC films with poor performance or, in other cases, important parameters such as the pumping energy fluence at the laser threshold could not be easily derived from the published data. Therefore, it is interesting to find a tool to compare the quality of different cell architectures in a systematic way. This is important to determine the most efficient strategies to obtain low threshold CLC lasers.

The ultimate target in the improvement of CLC-laser capabilities is to develop continuous wave (CW) lasers. The achievement of this objective presents two important difficulties. On the one hand the CLC materials cannot dissipate easily the energy flux due to the high pumping fluence required for lasing. On the other hand the existence of a triplet state in the dyes prevents to maintain laser action continuously. Only a work by Muñoz et al. [50] reported the fabrication of a CW laser by using a polymer-stabilized CLC with a pitch gradient across the cell thickness. However, the temporal coherence of the laser emission was very poor and laser emission was hardly distinguishable from amplified spontaneous emission (ASE). 

The main aim of this work is to analyze the different strategies used for the optimization of CLC laser cavities indicating some possible fields of improvement and pointing out the limitations of some of the strategies used so far. Firstly we will carry out comparative studies of different cell architectures from a theoretical point of view. Surprisingly, apart from studies in simple CLC cells [51,52,53,54], a detailed treatment of the laser characteristics in complex structures is still lacking in the literature and, up to now, only qualitative or semiquantitative aspects based on the concept of density of states (DOS) are normally considered. This problem will be treated in Section 2 using standard numerical methods, but incorporating some novel aspects in the calculations to properly quantify the performance of the different sample architectures. Next, the most promising cell designs will be experimentally tested to confirm the theoretical results. For this purpose eight different CLC samples will be thoroughly measured. In addition, we will present some new results about the prospects for CW lasers regarding the maximum achievable pulse width, and its dependence on the properties of the dye. Finally, we will draw some conclusions about the possibilities and limitations of the CLC lasers in general.

## 2. Theoretical Analysis of Different Cell Architectures

We carried out a study of different kinds of cells by using the 4 × 4 matrix transfer formalism developed by Berreman [55]. This approach can be applied to the case of stratified anisotropic media. We mainly focused on CLC-active cells sandwiched within two passive CLC layers acting as reflectors although other cell configurations were also studied. In our studies, the photonic band gap (PBG) of the two passive CLC layers overlaps the long-wavelength PBG edge of the active CLC layer in order to obtain the desired reflecting effect. We studied both the cases of reflectors with the same handedness as that of the active layer and with the opposite one. For the sake of completeness, the case of DML was also briefly treated. 

Under the Berreman method, the case of an amplifying medium can be implemented by using a complex dielectric tensor at each layer with a negative imaginary part. This imaginary part is achieved through the pumping, which gives rise to a population inversion in the dye molecules permitting then light amplification. We used an approximate expression for the local dielectric tensor of the form:

(1) $$\begin{array}{l} \varepsilon_{\left|\right|}\approx n_{e}^{2}\left(1-i\gamma_{\left|\right|}\right)\\ \varepsilon_{⊥}\approx n_{o}^{2}\left(1-i\gamma_{⊥}\right) \end{array}$$

where $\varepsilon_{\left|\right|}$ and $\varepsilon_{⊥}$ are the components of the dielectric tensor parallel and perpendicular to the local director for optical frequencies, $\gamma_{\left|\right|}$ and $\gamma_{⊥}$ are the corresponding ratios of the imaginary and real parts of the dielectric tensor, and $n_{e}$ and $n_{o}$ are the real extraordinary and ordinary refractive indices of the material. In the expression we have neglected the contribution of the extinction coefficient to the real part of the dielectric tensor. We have also assumed that the dispersion of the refractive indices in the optical frequency range is null. 

In our calculations we will assume that the gain anisotropy is similar to that of the absorption coefficients. This point can be justified since both the local absorption coefficient and the gain are connected to the orientation of the transition dipole moment of the dye in a similar fashion. For the calculations we will take the ratio $\gamma_{\left|\right|}/\gamma_{⊥}=3.5$ as in the case of the mixture of the classical nematic liquid crystal E7 and the dye 4-(dicyanomethylene)-2-methyl-6-(p-dimethylaminostyryl)-4H-pyran (DCM), in the proportion: 99:1 (wt %) at room temperature (see Appendix A). Thus, only one parameter, $\gamma_{\left|\right|}$, determines the amplifying features of the medium. 

**Figure 2:**
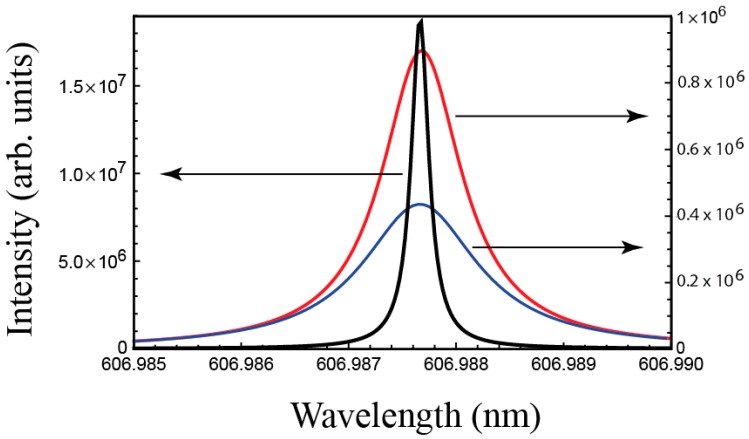
Emitted intensity spectrum of a simple CLC layer calculated for the first EM with three increasing $\gamma_{\left|\right|}$ values: 9.89 × 10^−4^ (red curve), 9.91 × 10^−4^ (black curve), and 9.93 × 10^−4^ (blue curve). Red and blue curves were represented in a different scale (right ordinate axis) for clarity.

Under amplifying conditions the reflectance R and transmittance T fulfill the condition T + R > 1. For a critical value $\gamma_{\left|\right|}^{th}$, the total output intensity diverges at a given wavelength. This is the situation for which laser emission occurs. For higher $\gamma_{\left|\right|}$ values, lasing does not occur anymore (see Figure 2). Therefore, $\gamma_{\left|\right|}^{th}$ is the parameter to be calculated in order to determine the lasing features of the sample. Belyakov and Semenov [56] obtained analytic expressions for the critical values $\gamma_{\left|\right|}^{th}$ corresponding to the different EM’s in the case of a simple CLC layer. However, under not too strong pumping conditions, lasing takes place only at the wavelength that presents the lowest $\gamma_{\left|\right|}^{th}$ value, so we will calculate exclusively this quantity. In our case, the complexity of the multilayer cells prevents us from obtaining simple analytic expressions for $\gamma_{\left|\right|}^{th}$ so we determined it numerically. The lasing wavelength was in all cases close to the long-wavelength edge of the PBG of the active medium. This was expected since we took $\gamma_{\left|\right|}>\gamma_{⊥}$. 

The calculation was performed under the condition that the incident and transmitted waves had the same polarization state. This provides two different solutions, i.e., two different eigenstates of transmission. Then, we used these eigenstates as input lights with intensity value equal to unity, and found the $\gamma_{\left|\right|}$ parameter that gives an output intensity spectrum characteristic of laser emission. We observed that only one of the two eigenstates promotes laser emission at a given wavelength. Hereafter we will refer to it as a laser eigenstate. 

**Figure 3:**
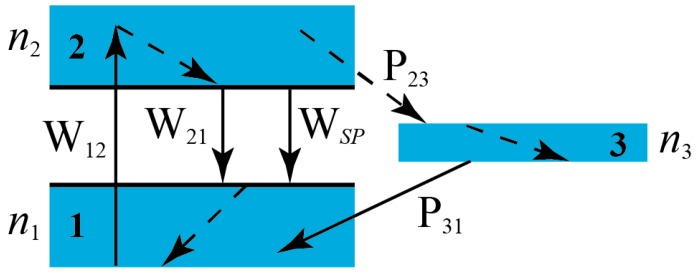
Scheme of the energy levels in a typical organic dye molecule. The arrows indicate different transitions in the excitation-deexcitation process. Dashed lines are purely non-radiative deexcitation.

Next we are going to relate $\gamma_{\left|\right|}^{th}$ with physical parameters easier to interpret. As previously mentioned, active media are obtained by doping CLCs with dyes. Typically, organic dyes present a structure of three effective electron bands as shown in Figure 3. Bands 1 and 2 are singlets and represent the ground and the excited states respectively. Number 3 is the ground triplet band. The densities of population of dyes at each band are denoted as *n*_1_, *n*_2_ and *n*_3_. Transitions represented with dashed lines inside the bands are very fast and purely non-radiative. As a consequence, population inversion takes place between the lowest energy level of band 2 and the highest energy level of band 1. $W_{12}$ and $W_{21}$ are the rates of induced transitions from 1 to 2 or 2 to 1, absorbing or emitting one photon respectively. $W_{sp}$ represents the rate of spontaneous transitions from level 2 to 1 by emission of one photon, and $P_{23}$ and $P_{31}$ are the rates of intersystem crossing processes between levels 2 and 3, or 3 and 1 respectively. Transition $P_{23}$ is also non-radiative.

When a dye-doped medium is pumped with an external source, a certain density of population in the excited state 2 is achieved. In this case, the medium can amplify an electromagnetic wave travelling through it due to stimulated emission. The transmittance corresponding to an eigenstate of the electromagnetic wave in the amplifying medium is given by:

(2) $$T=\exp\left(\sigma_{e}n_{2}L\right)$$

where *σ*_e_ is the emission cross-section of the dye for the corresponding polarization eigenstate, *L* is the sample thickness and *n*_2_ is the density of dye population in the excited state. When lasing takes place *n*_2_ remains almost constant. The corresponding value will be denoted as $n_{2}^{th}$. This quantity represents the density of population of excited dyes required for lasing and is a good indicator of the quality of the laser cavity, i.e., the lower the value of $n_{2}^{th}$ the better the quality of the cavity. Our main aim in our calculations was to determine this quantity for the different cell architectures. $n_{2}^{th}$ can be obtained from $\gamma_{\left|\right|}^{th}$ as follows: From Equation (1) the extraordinary complex refractive index ${\tilde{n}}_{e}$ for weakly amplifying media is given by:

(3) $${\tilde{n}}_{e}\approx n_{e}\left(1-i\frac{\gamma_{\left|\right|}}{2}\right)$$

On the other hand, comparing Equations (2) and (3) the following equation can be easily deduced:

(4) $$\frac{\sigma_{e}n_{2}L}{2}=\frac{2\pi n_{e}}{\lambda }\frac{\gamma_{\left|\right|}}{2}L$$

where *λ* is the vacuum wavelength of the electromagnetic field that is amplified. Therefore, it is straightforward to obtain $n_{2}^{th}$ by using Equation (4) for lasing conditions, i.e.,

(5) $$n_{2}^{th}=\frac{2\pi n_{e}\gamma_{\left|\right|}^{th}}{\sigma_{e}\lambda }$$

where *λ*, in this case, is the laser wavelength. Although $n_{2}^{th}$ does not determine completely the threshold pumping energy required for lasing, since it takes into account neither the dynamical aspects of the laser process nor the way the pumping light is absorbed, it is useful for comparing the quality of the different cell architectures to each other. 

For the simulations we use the optical data listed in Table 1. These parameters are typical of CLC’s and are, in fact, quite similar to those of one of the compounds we have studied experimentally (see Section 3).

The different types of cells we have examined will be denoted as follows: AL5 or AL10 means simple left handed active CLC samples of 5 and 10 μm thickness respectively. In our study, the passive reflectors thickness is in all cases 5 μm and the helical pitch 370 nm. We have examined left and right handed passive CLC layers that will be denoted as PL and PR. According to this nomenclature, for example, an active layer of 5 μm thickness sandwiched within two left-handed reflectors is denoted as PLAL5PL.

**Figure 4:**
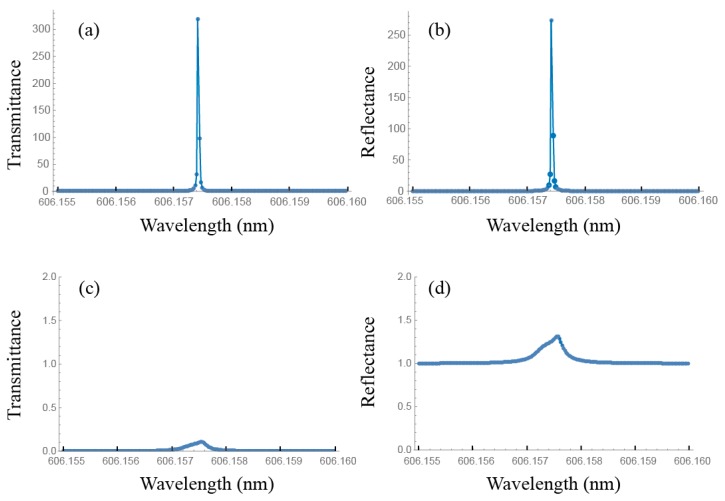
Transmittance (**a**) and reflectance (**b**) versus wavelength for the lasing mode of the lowest threshold gain for the PLAL10PL sample; (**c**,**d**) are the transmittance and reflectance for the non-diffracting eigenstate under the same gain conditions.

As an example we show in Figure 4 the emission spectrum corresponding to the cell PLAL10PL. Figure 4a,b show the transmitted and reflected intensity spectra for the laser eigenstate. They represent the emission intensities that emerge by both external faces of the cell. In general the polarization eigenstate is elliptically polarized. When lasing takes place, both intensities are almost equal due to the symmetry of the cell. It is remarkable that the laser emission wavelength corresponds to that of the low energy edge of the PBG of the active layer. As the laser line is very narrow, only a small range of emission wavelengths is depicted. Figure 4c,d are obtained with the non-diffracting eigenstate input. In this case, no lasing is observed. Our calculations also permit to obtain the polarization of the light eigenstates inside the sample. We have observed that the polarization of the laser eigenstate in the active layer is linearly polarized along the local director. 

Table 2 gathers the results for $\gamma_{\left|\right|}^{th}$ and $n_{2}^{th}$ in different types of cells. $n_{2}^{th}$*/N* (%) is the percentage of the dye population in the excited state required for laser emission relative to the total number of dye molecules. In the case of simple cells approximate analytical expressions for $n_{2}^{th}$ have been given in the literature [56,57]:

(6) $$n_{2}^{th}\approx \frac{2p^{2}}{\sigma_{e}L^{3}}{\left(\frac{n_{e}^{2}+n_{o}^{2}}{n_{e}^{2}-n_{o}^{2}}\right)}^{2}$$

According to the results in Table 2 it is clear that the use CLC passive layers acting as reflectors improves very efficiently the quality of the laser cavity. In fact, the $n_{2}^{th}$ values are reduced in several orders of magnitude respect to the case of the corresponding CLC active sample without reflectors. Some of these figures for $n_{2}^{th}$ cannot be reached with simple cells unless unrealizable thicknesses or birefringences are assumed (see Equation (6)). The complex cells with CLC reflectors, with the same handedness as that of the active layer, present the highest quality. In fact, the case of PLAL10PL presents a $n_{2}^{th}$ value so small that laser is expected to be almost thresholdless.

In the case of CLC passive reflectors with opposite handedness as that of the active layer, it is clear that the improvement of the quality of the laser cavity is worse. This point is easy to understand since in this case, the reflectors act as in a Fabry-Perot for the circular right-handed polarization state. Therefore, the active CLC layer does not play the role of an advantageous photonic structure. In this respect, a smaller $n_{2}^{th}$ would be expected if the active layer were replaced simply by an isotropic material, since, in this case, losses would be smaller. The irrelevance of the active photonic structure is evident in our results since the quality of the laser cavity is similar in the cases of PRAL5PR and PRAL10PR.

We have also considered the case of complex cells in which the different layers are separated by thick glasses (~2 mm). These cell arrangements are interesting in practice because they can be constructed very easily by simply stacking different standard glass cells that contain the required CLC structures. In all cases the modes corresponding to the lowest $n_{2}^{th}$ present similar values to those of cells without the glasses.

**Figure 5:**
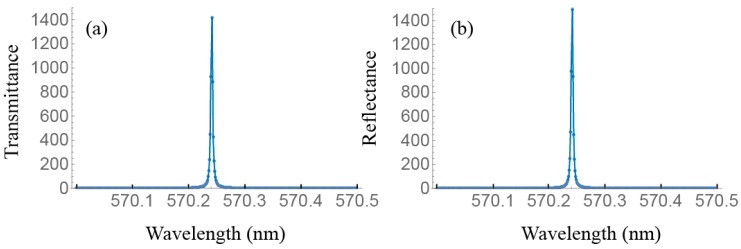

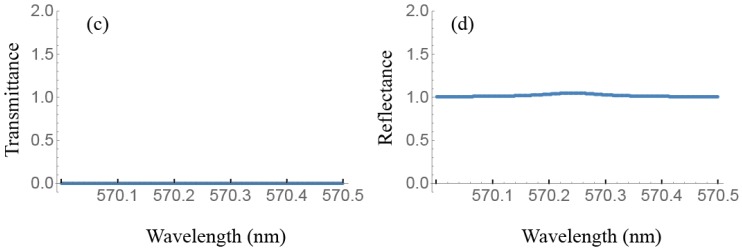
Transmittance (**a**) and reflectance (**b**) versus wavelength for the lasing mode of the lowest threshold gain of the DML10 sample; (**c**,**d**) are the transmittance and reflectance for the non-diffracting eigenstate under the same gain conditions. A phase jump of π/2 in the twisting angle was considered in the middle of the cell.

We finally treat the case of DML, where a phase-jump in the twisting angle of the LC director appears at a certain point of the helical structure, as in Figure 1a. According to the reported experimental results [42,43], laser emission corresponding to these modes presents a lower threshold than that of the EM of the non-distorted structure. In practice, this kind of defects can be induced by coupling two active CLC layers, usually polymeric structures, with a controlled phase-jump in the LC director at the coupling interface. Following the Berreman formalism, as in the previous cases, we have studied the gain values required for laser emission corresponding to the defect modes. We have observed that the laser wavelength depends on the phase jump angle of the defect and the corresponding $n_{2}^{th}$ value is somewhat smaller than that of the PBG edge mode. In the case of a left handed CLC sample of 10 μm thickness with a defect mode of angular jump of π/2 in the middle of the sample (DML10) (see Figure 1a) the gain value is about one third the value of the edge mode of the non-distorted structure (last line in Table 2). Figure 5a,b show the transmitted and reflected intensity spectra of the laser mode and Figure 5c,d that of the non-diffracting eigenstate. It is remarkable that the polarization of the laser is right-handed elliptical. For a sample of 20 μm of thickness with a similar defect, the polarization is roughly right handed circular in agreement with the results obtained by Schmidtke et al. [42]. The laser wavelength due to the defect mode can be tuned all over the entire range of the PBG by changing the angular jump from 0 to π. In the case of a phase jump of π/2, laser takes place at the wavelength just in the middle of the PBG.

In view of the general trend of the results in Table 2, one could draw the conclusion that a higher level of sophistication in the structures of the laser cavities could lead eventually to a situation of thresholdless lasing, which would be appropriate for designing CW lasers. However, we will see now that the expression for $n_{2}^{th}$ must be corrected in a non-trivial fashion to give account of the losses in the cavity due to light scattering or absorption. 

In principle, $n_{2}^{th}$ is related to the dwelling time *τ_c_* of laser photons in the cavity in the absence of losses by means of the expression [57]:

(7) $$n_{2}^{th}=\frac{n}{c\sigma_{e}\tau_{c}}$$

where *n* is an average refractive index and *c* the speed of light in vacuum. These are the $n_{2}^{th}$ values shown in Table 2. Nevertheless, Equation (7) and our calculations disregard the pernicious contribution of the losses in the cavity (scattering and absorption) that are always present in CLC materials. If we take into account these losses, the expression for $n_{2}^{th}$ is corrected as [54]:

(8) $$n_{2}^{th}=\frac{1}{\sigma_{e}}\left(\beta +\frac{n}{c\tau_{c}}\right)$$

where *β* is the so-called coefficient of distributed losses. We will see that for very high-quality laser cavities, as is the case of complex layers with passive reflectors, the lowest limit for $n_{2}^{th}$ is almost exclusively determined by the value of *β*. As a numerical example we estimate the case of the cell PLAL5PL. From Table 2 and Table A1, *n/cτ_c_* = $n_{2}^{th}$*σ_e_* = 1.6 cm^−1^. As will be shown below, this quantity is much smaller than typical *β* values, which are in the range of 100 cm^−1^ (see Appendix A and Section 3 for experimental *β* values). Then, from Equation (8), we deduce that it is pointless to try to reduce the “theoretical” $n_{2}^{th}$ by means of complicated cavity designs, because, in practice, $n_{2}^{th}$ is limited by the scattering and absorption losses. The simple consideration of the molecular orientation fluctuations, intrinsic in mesogenic phases, already gives rise to enough scattering of light so as to spoil any “highly-developed” CLC laser cavity. We will come back to these ideas in the experimental part of the paper. 

We finish this section by giving a link between $n_{2}^{th}$ and the threshold energy per pulse *E_th_* in order to compare the previous theoretical analysis with experimentally measurable quantities. In the case of nanosecond or picosecond pump pulses, an approximate analytical expression for *E_th_* was obtained by Sanz-Enguita et al. [10]

(9) $$E_{th}=2h\nu_{a}SLn_{2}^{th}\frac{1+0.53\Delta \tau_{p}\left(P_{23}+1/\tau_{f}\right)}{1-\exp\left(-\sigma_{a}n_{1}^{th}L\right)}$$

where *h* is Planck’s constant. This equation was deduced from the kinetic equations of the excited state populations of dyes and emitted light developed by Shtykov and Palto [40]. In Equation (9) *υ_a_* is the pumping light frequency, *S* is the pumped area, *L* the active sample thickness, ∆*τ_p_* is the pumping pulse duration, *τ_f_* is the fluorescence lifetime, *σ_a_* is the absorption cross-section for the pumping radiation, and $n_{1}^{th}$ the dye population density in the ground level at the threshold. The expression can be applied to any laser cavity provided that the energy level scheme of the dyes is that of Figure 2. *E_th_* is roughly proportional to $n_{2}^{th}$, as expected. This equation will be used to account for the results obtained in the next experimental section in which some CLC simple cells will be studied together with the complex cells that are expected to present the lowest *E_th_*, i.e., the cases of cells PLAL5PL and PLAL10PL. The experiments will be carried out using two different dyes.

## 3. Experimental

### 3.1. Sample Preparation and Experimental Setup

Two CLC active mixtures were prepared. Both of them were based on E7 (Synthon) and the chiral twisting agent D* (compound 2 in reference [58]). The first material (denoted by DCM) contained the dye of the same name, and the second material (PM) was doped with 1,3,5,7,8-pentamethyl-2,6-di-t-butylpyrromethene-difluoroborate complex (PM597). The proportion of each component (in wt %) is shown in Table 3.

Apart from these materials another passive mixture was also prepared in a similar way. This mixture has no dye, and was chosen in such a way that the long wavelength side of the photonic band-gaps of the above DCM and PM materials were contained within its reflection band. The idea was to use this material to construct a passive CLC reflector to enhance the laser emission of a simple active CLC cell, as has been explained in Section 2. Its spectral characteristics are also included in Table 3 under the label “passive mirror mixture”.

**Figure 6:**
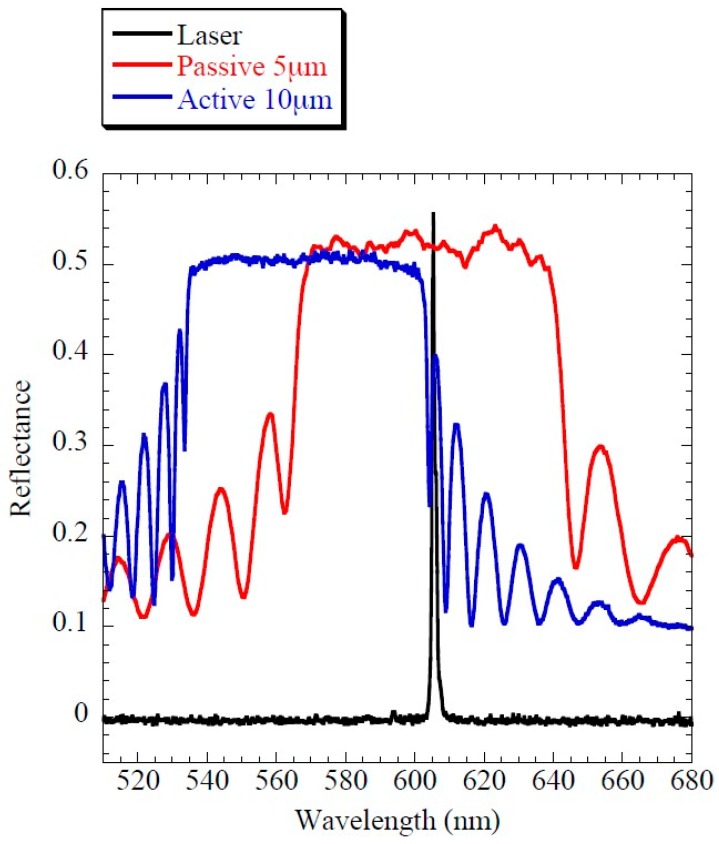
Reflectance and laser emission spectra of the CLC cells used in the experiments. In the case of the active cell, the reflectance measurements were carried out before adding the dye.

The band gaps of the CLC materials were characterized by measuring their reflectance spectra with a fiber-based spectrometer (Avantes). The materials were aligned in the Cano geometry (helix perpendicular to the cell substrates) using planar cells of 5 and 10 μm of thickness for the passive and active cells respectively. An example of the reflectance spectra of one of the dye-doped CLC cells and the passive-mirror material is shown in Figure 6. In the figure the emission of the CLC laser is also depicted.

The laser generation and performance were studied in two types of cavity architectures: simple cells and complex resonator cells. The influence of the thickness of the active CLC layer was also investigated, analyzing two thicknesses: 5 μm and 10 μm. The resonators were constructed by sandwiching an active simple cell between two 5 μm passive mirrors. A microscope immersion-oil drop was deposited on the interface of the different glass cells, and the whole assembly was firmly pressed with screws. 

A total of 8 cells were studied. We will denote the simple cavities by the name of the material (DCM or PM) followed by a number equal to its thickness in μm. The resonator cells will be designated by adding the letter R in front of the name of the middle cell. For example, DCM10 and R-PM5 are a simple 10 μm active cell of DCM and a resonator cell with a middle 5 μm active cell of PM respectively.

**Figure 7:**
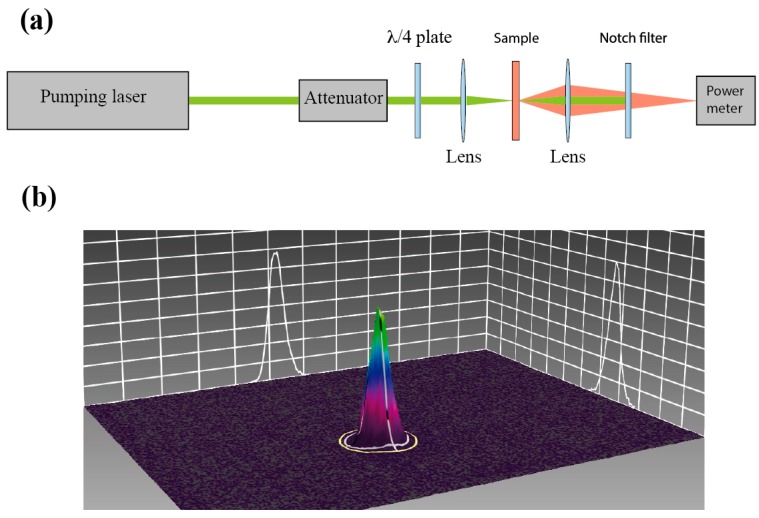
(**a**) Experimental setup used to characterize the laser performance of the different cell architectures; (**b**) Beam profile of the pumping laser on the sample. As can be seen the spot roughly presents a Gaussian intensity distribution.

Figure 7a shows the experimental setup. Cells were optically pumped using an Nd:YAG laser operating at the second-harmonic frequency (wavelength 532 nm). The light polarization was circular with opposite polarization sense to that of the CLC helix in order to optimize the excitation conditions. The laser emitted 14 ns-long pulses with a repetition rate of 5 Hz, and was focused on the samples at normal incidence by using a lens of 20 cm of focal length. The spatial profile of the energy distribution of the laser spot on the sample was measured with a CCD camera (Ophir). As can be seen (Figure 7b) the spot is near Gaussian, with a diameter D4σ = 240 μm (FWHM = 144 μm). The light emitted by the CLC cells was focused with a lens of 5 cm of diameter onto a power meter (Ophir). A notch filter for 532 nm was placed behind the sample to remove the pumping light. To analyze the spectrum of the emitted light the power meter was substituted by an optical-fiber spectrometer (AvaSpec 2048).

### 3.2. Fits of the Laser Emission Curves

**Figure 8:**
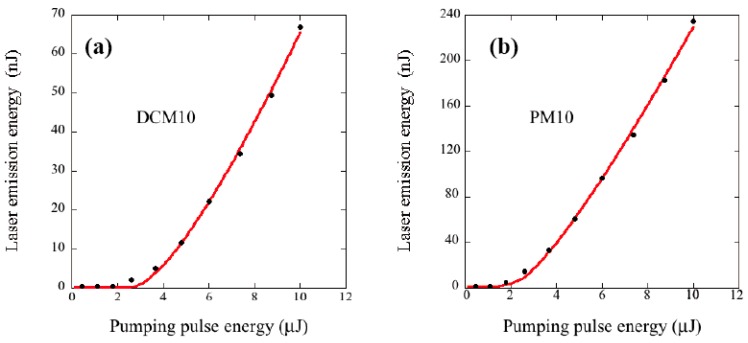

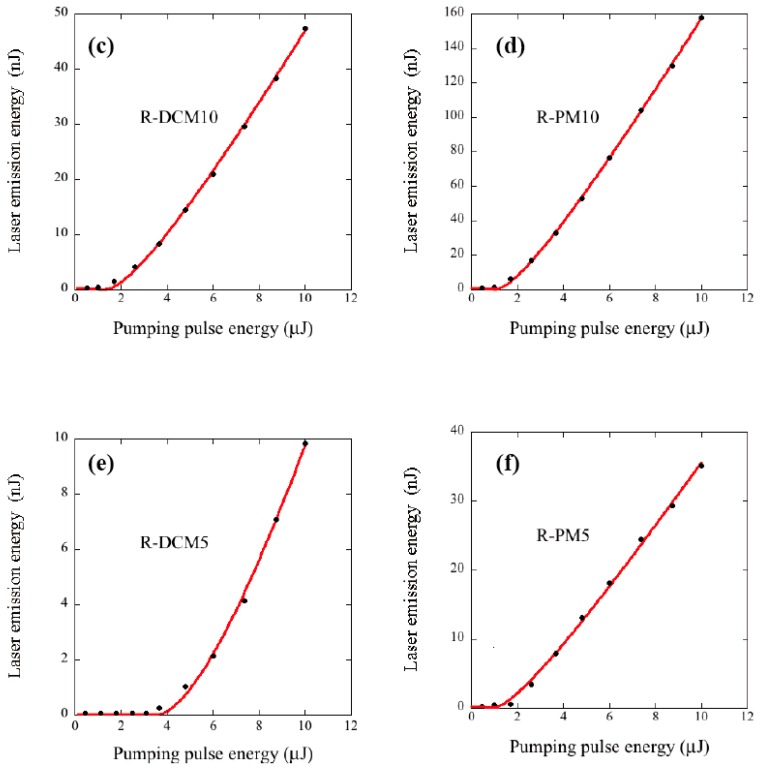
Laser emission energy as a function of pumping excitation energy for the different samples studied (**a**–**f**). Continuous lines are fits to Equation (14).

Figure 8 shows the dependence of the laser emission energy on the excitation energy for different cell architectures and for DCM and PM597 dyes. We have not included the curves for the simple cells of 5 μm because lasing was not possible in either of them. In all cases the fluorescence contribution (at low excitation energies) was subtracted from the total signal using linear extrapolation.

The threshold energy per pulse *E_th_* needed to achieve lasing and the slope efficiency *η* were obtained from a fit of the experimental curves to a linear law of the type

(10) $$I_{out}=\eta \left(I_{in}-I_{th}\right)$$

where *I_th_* is the threshold intensity, and *I_in_* and *I_out_* are the pumping input and output laser intensities respectively. In the case of pulsed pump sources all intensities *I_th_*, *I_in_* and *I_out_* can be considered intensities averaged over time.

**Figure 9:**
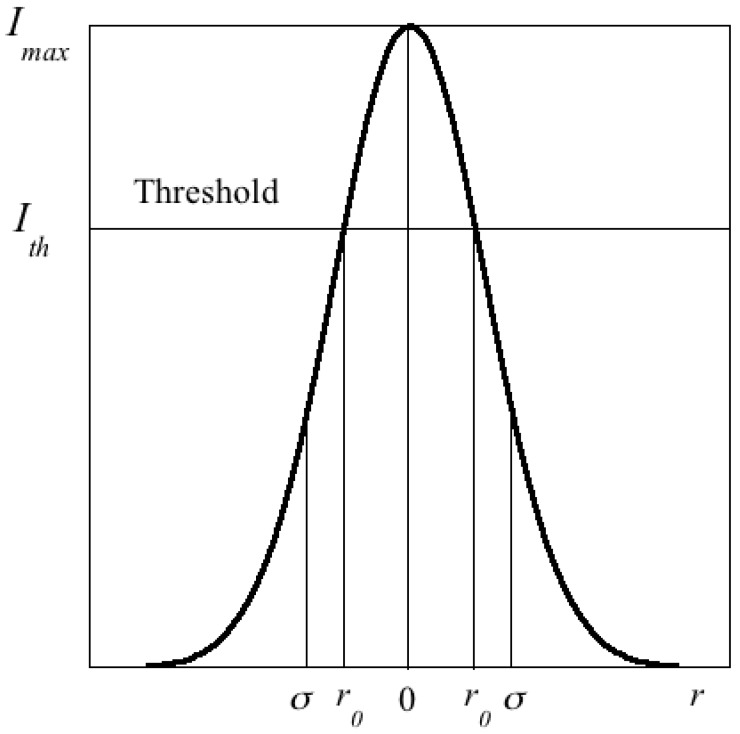
Scheme of the spatial distribution of the pumping intensity in a Gaussian spot. Lasing is only possible within a circle of radius *r*_0_.

It is interesting to note that Equation (10) cannot be directly extrapolated to a law involving the input and output energies per pulse, *E_in_* and *E_out_*, unless the shape of the illumination spot from the pumping source is top hat-like. In the latter case we obviously conclude $E_{out}=\eta \left(E_{in}-E_{th}\right)$ from (10) but, in general, this expression is not strictly valid although it is commonly used in the literature. The problem occurs because for a rounded Gaussian-type spot, not all the energy contained in the spot contributes to generate laser light. With reference to Figure 9, only the energy deposited on the sample within a circle of radius *r*_0_ gives rise to the lasing process. Therefore, assuming a Gaussian spot of maximum (time-averaged) intensity *I_max_* and width *σ*,

(11) $$I\left(r\right)=I_{\max}\exp\left(-r^{2}/\sigma^{2}\right)$$

We deduce by integration of (10),

(12) $$E_{out}=(\eta /f)\int_{0}^{r_{0}}\left[I\left(r\right)-I_{th}\right]2\pi rdr$$

where *f* is the pulse repetition rate of the pumping source. On the other hand, the total energy per pulse supplied to the sample is given by

(13) $$E_{in}=(1/f)\int_{0}^{\infty }I\left(r\right)2\pi rdr=I_{\max}\pi \sigma^{2}/f$$

Finally, carrying out the integration in Equation (12) and using (13) we obtain

(14) $$E_{out}=\eta \left[E_{in}-E_{th}\left(1+\ln\frac{E_{in}}{E_{th}}\right)\right]$$

with $E_{th}=I_{th}\pi \sigma^{2}/f$.

Equation (14) is thus the translation of Equation (10) from intensities to energies per pulse in the case of Gaussian spots. The logarithmic term brings about a progressive increase of the slope *dE_out_/dE_in_* from 0 to *η*. In general, *dE_out_/dE_in_* = *η* (1 − *E_th_/E_in_*), and only if *E_in_* >> *E_th_* the slope of the curve is a constant and equal to *η*.

Continuous lines in Figure 8 are fits to Equation (14). The resulting parameters and their errors are collected in Table 4. The slopes were doubled to take into account the laser emission from both sample faces.

Some interesting features can be noticed:

(i) The resonator architecture clearly lowers the *E_th_* value. The improvement is especially noticeable for the cells of 5 μm, which do not show laser emission (*E_th_* = ∞) in the configuration of simple cells.
(ii) The improvement obtained for *E_th_* with the incorporation of the resonator is the better when the starting point (simple cell) is the worse. For example, the threshold of the best simple cell (PM10) only decreases by a factor 1.4 in the resonator configuration whereas *E_th_* of R-DCM10 represents an improvement from DCM10 by a factor 2.3.
(iii) There seems to be a limit for *E_th_*, below which we cannot go. In our case, *E_th_* ≈ 0.8 μJ. Since the spot of the incident light has a width *σ* = 85 μm ($\sigma =\frac{D4\sigma }{2\sqrt{2}}$), this implies it is necessary a minimal fluence of *E_th_*/π*σ*^2^ = 3.5 mJ/cm^2^ to achieve laser generation. The existence of this limit is caused by the effect of the distributed losses in the laser cavity. From Equation (8) it can be seen that a non-null *β* establishes a limit for $n_{2}^{th}$ = *β*/$\sigma_{e}^{\left|\right|}$, irrespective of the *τ_c_* value. Since *E_th_* is roughly proportional to $n_{2}^{th}$ (see Equation (5)), this implies it is the scattering (or other kind of cavity losses such as absorption) the ultimate responsible for a minimum *E_th_*.
(iv) It seems there is no clear improvement for *η* when the complex resonator architecture is used (a trivial exception to this rule is the case of the 5 μm cells, which did not show laser emission in the absence of passive reflectors). Though we have not gone further into this point, it seems likely that the reduction of the thickness of the passive reflectors can give rise to a growth of *η*. This hypothesis is based on the idea that the passive reflectors play a role similar to the exit mirrors of a Fabry-Perot cavity, and in this configuration there exists an optimum reflectance that maximizes the laser output power. Thus, if the reflectance is too high, the slope efficiency starts to reduce (evidently, in the limiting case of a reflectance equal to unity the slope efficiency is zero). Presumably the thickness of the passive mirrors actually used in our cells is well above the optimum value. Some experimental studies about the optimum thickness of these types of reflectors can be found in Zhou et al. [49].

We now turn to further develop some of these ideas by analyzing more quantitatively the threshold energies. For the simulations we used the material parameters listed in Table 5. These parameters were taken from the literature and from our own measurements (see Appendix A). The resulting theoretical *E_th_* values are shown in Table 4 in parenthesis. The data were obtained following the procedure indicated in Section 2 to first get $n_{2}^{th}$, then include the *β* contribution from Equation (8), and finally use the approximate formula Equation (9). As can be seen, the agreement with the experiments is in general good, although deviations are found for the thicker cavities of DCM. We do not know the reason for these discrepancies but, certainly, some of spectroscopic parameters of Table 5 must be affected of relatively large uncertainties. Other parameters that give rise to further difficulties are the scattering losses, which show changes across the cell surfaces. In fact, experimental *E_th_* was found to vary depending on the illumination point on the cavity surfaces, especially for the complex resonator cells. Moreover, in those cases the assembly of the passive mirrors is slightly different each time, which can produce uncontrolled additional losses at the interfaces of the different components (due e.g., to misalignments or lack of parallelism between the surfaces). These additional losses were modeled by adding a global loss factor *β*’ = 100 cm^−1^ for the complex cells. Taking all these factors into account we conclude that Equation (9) for *E_th_* describes reasonably well the behavior of the different laser cavities and materials studied.

### 3.3. Continuous-Wave Lasing in CLC’s

Up to now we have dealt with lasers pumped by pulsed sources in the range of nanoseconds. However, it is interesting to study the performance of these systems in other time ranges. In particular, it is especially appealing to get CW lasing in CLC cavities. This goal has not been achieved so far, though there have been some claims in this respect. The difficulties are the following:

As can be seen from Equation (9) *E_th_* grows with the pulse width ∆*τ_p_*. Evidently that equation does not hold in the limit of CW regime, but fortunately that case has been explicitly worked out in ref. [57] for a two-level system. The resulting threshold intensity *I_th_* is

(15) $$I_{th}\approx n_{2}^{th}hcL/\lambda \tau_{f}$$

Taking typical data *L* = 10 μm, *τ_f_* = 2 ns, *λ* = 600 nm $n_{2}^{th}$ ≈ *β*/$\sigma_{e}^{\left|\right|}$ ≈ 0.15 × 10^19^ cm^−3^, we get *I_th_* = 280 kW/cm^2^, i.e., a power of 60 W should be focused within a spot of σ= 80 μm to produce lasing. In view of these high powers it seems likely that CW lasing must presumably be accompanied by high local temperatures that can significantly alter the quality of the cavity.

Even if some dissipation mechanism is implemented to avoid the above effect there is another still more serious problem: If a conventional organic dye is used, there is a triplet state whose effect is extremely pernicious for lasing using pumping sources with large pulse widths. Equation (15) is not valid in this case because of the existence of the triplets, which eliminates molecules from the lasing channel. The excited molecules at level 2 go to the triplets with rate *P*_23_. Therefore, after long enough time the laser channel will be completely empty, killing the laser emission. In addition, the dye population in level 3 can contribute to increase the heat generated on the sample by means of absorption of the pumping radiation. Starting from the triplet level, the molecules are promoted by photon absorption to upper levels, which finally deexcite down to the ground level 1 through mainly non-radiative processes. Only if *P*_23_ is small, *P*_23_ << 1/∆*τ_p_*, the effect is not important, since for such pumping pulse durations the molecules at level 2 do not have time to go to the triplet in a significant proportion. In the case of our dyes, *P*_23_ ≈ 10^7^–10^8^ s^−1^ (see Table 5). Thus, we expect problems for ∆*τ_p_* > 10^−7^ s. We have carried out simulations by solving numerically the dynamic equations of ref. [54] allowing *P*_23_ to vary. We have found that a sufficiently large *P*_23_ can kill the lasing for any pulse width ∆*τ_p_*, irrespective of the pumping intensity. 

**Figure 10:**
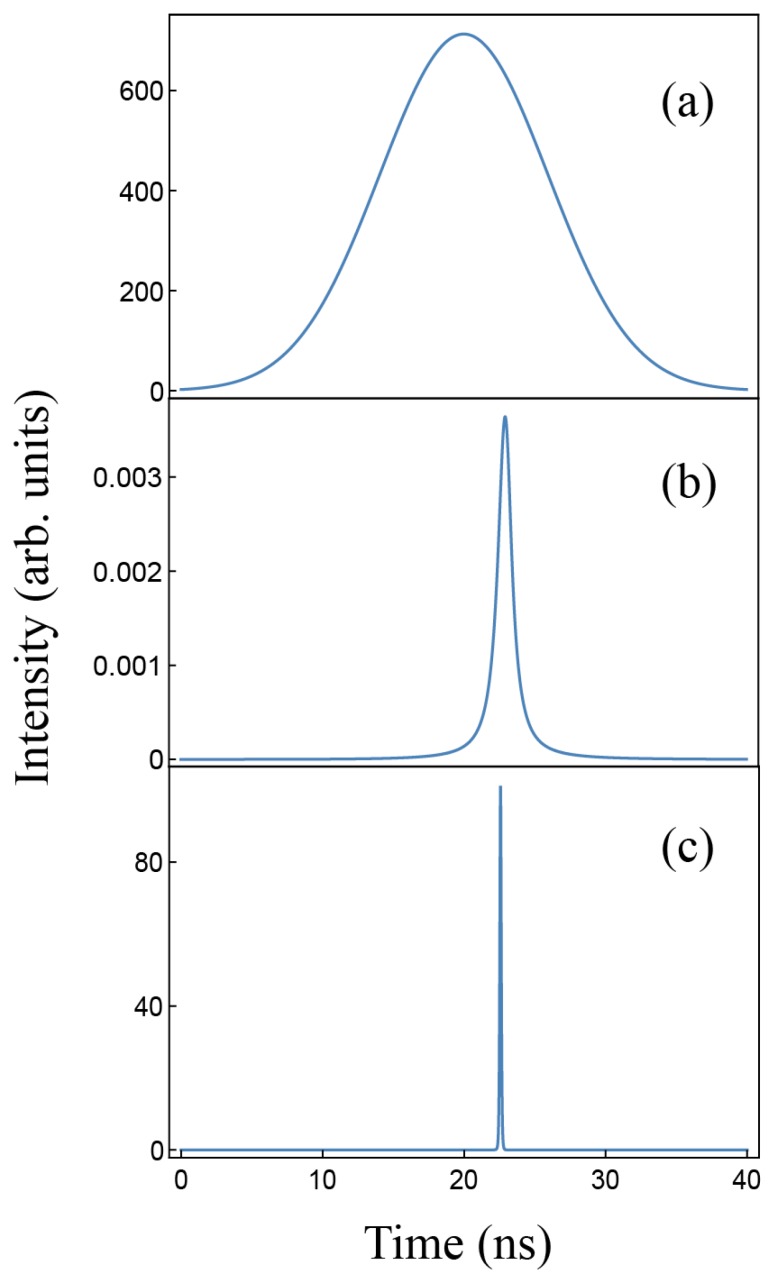
Temporal profile of a 14 ns pulsed pumping source (**a**); and calculated light intensity emitted in a situation of fluorescence emission (**b**); and laser emission at the threshold (**c**); using the data of Table 5 for PM597. Arbitrary units for curves (**b**,**c**) are the same.

Figure 10 shows an ideal pumping temporal profile (a), and the corresponding output light in a situation of fluorescence (b) and laser emission at the threshold (c). The lasing onset is easily identified because the emitted pulse suddenly becomes very narrow and increases its size significantly when the threshold is approached.

**Figure 11:**
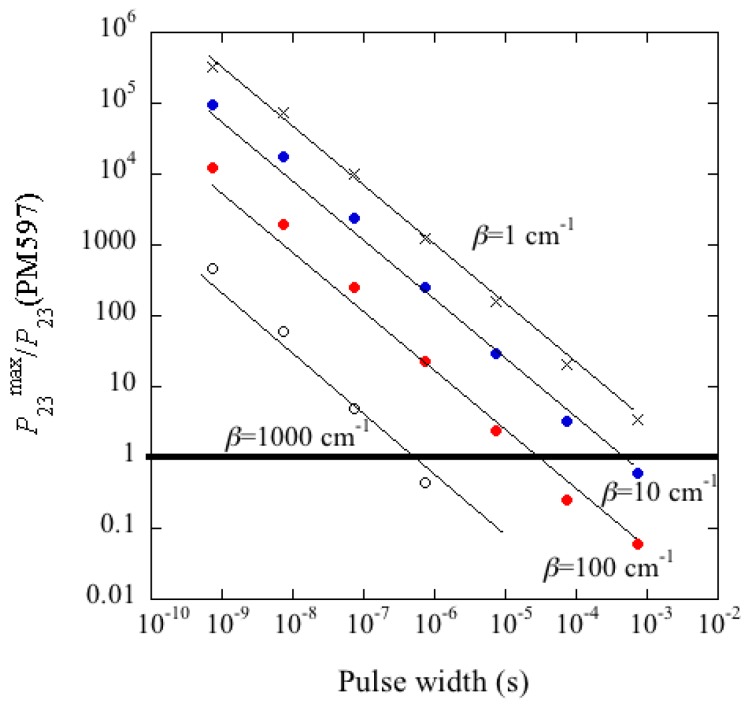
Maximum *P*_23_ values allowing for lasing action in a 13 μm thick CLC sample doped with PM597 (1 wt %) as a function of the pumping pulse width. *P*_23_ values are scaled relative to the actual *P*_23_ value for PM597. The different symbols correspond to different *β* values. Continuous lines are fits to a law $P_{23}^{\max}\approx A/{\left(\Delta \tau_{p}\right)}^{\delta }$.

The results of the simulation are shown in Figure 11. The spectroscopic data were those of PM597 (see Table 5) except *P*_23_ and *β*, which were allowed to vary in the calculation process. It was supposed 1/*τ_c_* = 0, i.e., we assumed a laser cavity just limited by a non-null *β* (as in our resonator-type cells). In the calculations the pumping intensity was increased until threshold was reached for given values of ∆*τ_p_* and *P*_23_. Then, a maximum *P*_23_ was obtained above which lasing does not occur for any pumping intensity. In the plot, the maximum *P*_23_ that permits lasing is displayed versus the pumping pulse width for different *β*. The horizontal line corresponds to the actual *P*_23_ value of PM597. Therefore, for a given *β* lasing takes place in the ∆*τ_p_* range that corresponds to points above this horizontal line. For example, if *β* = 100 cm^−1^, lasing is only possible for ∆*τ_p_* < 10^−5^ s. As can be seen, a small *β* increases the ∆*τ_p_* range substantially, but even for unrealizable tiny *β* values CW lasing is impossible.

According to our simulation, the maximum *P*_23_ follows a law $P_{23}^{\max}\approx A/{\left(\Delta \tau_{p}\right)}^{\delta }$, where *δ* ≈ 0.8 in all cases, and *A* is a value dependent on *β* (continuous lines in Figure 11). Then, if we want ∆*τ_p_* = ∞, this implies $P_{23}^{\max}$ ≈ 0, i.e., no triplet state is allowed in a dye for a CW laser. This excludes all the organic dyes commonly used in CLC lasers. To achieve CW lasing, an alternative to explore is the substitution of organic dyes by quantum dots (QD’s), which behave as two-level systems. QD’s can be integrated within CLC’s if they are functionalized and, in fact, the possibility of lasing has already been demonstrated [17]. 

## 4. Concluding Remarks

In this paper, we have analyzed the main strategies proposed in the literature to optimize the performance of CLC lasers. We have seen that the use of a variety of complex structures (resonators, defect-mode lasers and other multilayer designs) can theoretically give rise to huge improvements in *E_th_* in comparison with those of simple cells. In particular, the three-layer structure constituted by an active cell sandwiched between two cholesteric mirrors of the same chirality as the active part permits to reach a virtually thresholdless lasing. However, this improvement is not realizable in practice, but is limited by the existence of scattering and absorption losses. In CLCs, even assuming a perfect quality of the sample, the fluctuations of the director orientation already give rise to such large light scattering that the upgrades achieved by the sophistication of the cavities are greatly spoiled. In real cells the problem is even worse because the defects in the sample alignment and imperfections produce more scattering. The only possibility for improvement is then to optimize the alignment quality (to decrease *β* as much as possible) or to raise the parameter $\sigma_{e}^{\left|\right|}$, which also reduces the limit value for $n_{2}^{th}$ (this limit is given by *β*/$\sigma_{e}^{\left|\right|}$, see Equation (8)). The latter point seems to be absolutely essential, and implies to draw our attention to the dyes instead of to the CLCs. In this respect, fluorescent molecules with high emission cross-sections would be needed and, if possible, they should be easily integrable within the mesogenic matrix. In addition, the resulting mixtures should present high values of the dye order parameter (see Equation (A3)). On the other hand, dyes showing low quantum yields to triplet states are required for CW lasing. It has been shown that the existence of the triplet population prevents lasing above a certain pumping pulse width. To solve this problem a possibility to explore is the use of QD as fluorescent agents, since they behave as two-level systems. An important disadvantage of these systems is their null order parameter in the liquid crystal matrix. This implies relatively high threshold energies, at least for the materials examined so far. We expect that more efficient organic dyes or QD’s can improve the CLC laser performance, and this is our research objective for the near future.

## Figures and Tables

**Table 1:** Optical parameters used in the simulation for the active CLC (cholesteric liquid crystals) material. The density of dye molecules *N* corresponds to a proportion of 1 wt % of the DMC dye in an E7 matrix. *p* denotes the helical pitch.

*n*_e_	*n*_o_	*σ*_e_	*N*	*p*
1.72	1.52	1.53 × 10^−16^ cm^2^	1.8 × 10^19^ cm^−3^	350 nm

**Table 2:** Gain thresholds and percentages of excited dye molecules required for lasing in the different types of samples analyzed in the simulations.

Type of CLC Sample	$\gamma_{\left|\right|}^{th}$	$n_{2}^{th}$/*N* (%)
AL5	6.3 × 10^−3^	44
AL10	9.9 × 10^−4^	6.5
PLAL5PL	9.5 × 10^−6^	0.06
PLAL10PL	6.4 × 10^−7^	0.004
PRAL5PR	4.0 × 10^−5^	0.3
PRAL10PR	5.0 × 10^−5^	0.36
DML10	3.3 × 10^−4^	2.0

**Table 3:** Composition and band-gap range of the studied CLC materials.

Material	E7	D*	Dye	Reflection Band (nm)	Helical Pitch (nm)
DCM	93.9%	5.2%	0.9%	533–605	352
PM	93.3%	5.8%	0.9%	519–590	343
Passive Mirror Mixture	95.2%	4.8%	0%	564–642	371

**Table 4:** Experimental threshold energies *E_th_* and slope efficiencies *η* for the different laser cavities studied. In parenthesis the theoretical *E_th_* values according to Equation (9).

Cell	R-DCM10	DCM10	R-DCM5	DCM5	R-PM10	PM10	R-PM5	PM5
*E_th_* (μJ)	1.06 ± 0.08 (2.8)	2.4 ± 0.2 (4.4)	3.2 ± 0.2 (3.6)	∞ (∞)	0.88 ± 0.04 (0.70)	1.2 ± 0.1 (1.02)	0.82 ± 0.06 (0.73)	∞ (∞)
*η* (%)	1.42 ± 0.05	3.1 ± 0.2	0.64 ± 0.04	----	4.5 ± 0.1	7.2 ± 0.4	1.00 ± 0.03	----

**Table 5:** Parameters used for the simulations. The pumping wavelength was 532 nm and the laser emission wavelengths were 605 nm and 590 nm for the materials with dyes DCM and PM597 respectively. $\sigma_{a}^{CL}$ is the absorption cross-section for the pumping light.

Material	DCM	PM
Pitch (nm)	352	343
*n*_e_, *n*_o_	1.73, 1.52	1.73, 1.52
*τ_f_* (ns)	1.2 ^a^	4.0 ^d^
*P*_31_, *P*_23_ (s^−1^)	10^4 a^, 5 × 10^7 a^	10^4 a^, 2.3 × 10^6 e^
$\sigma_{e}^{\left|\right|}$, $\sigma_{a}^{CL}$ (cm^2^)	1.53 × 10^−16 b^, 0.62 × 10^−16 c^	1.44 × 10^−16 d^, 2.3 × 10^−16 c^
*β* (cm^−1^)	150 ^c^	150 ^c^
*S* (μm^2^)	4.5 × 10^4^	4.5 × 10^4^
*N* (cm^−3^)	1.8 × 10^19^	1.46 × 10^19^

^a^ ref. [54], ^b^ ref. [52], ^c^ Appendix A, ^d^ ref. [59], ^e^ ref. [60].

**Figure A1:**
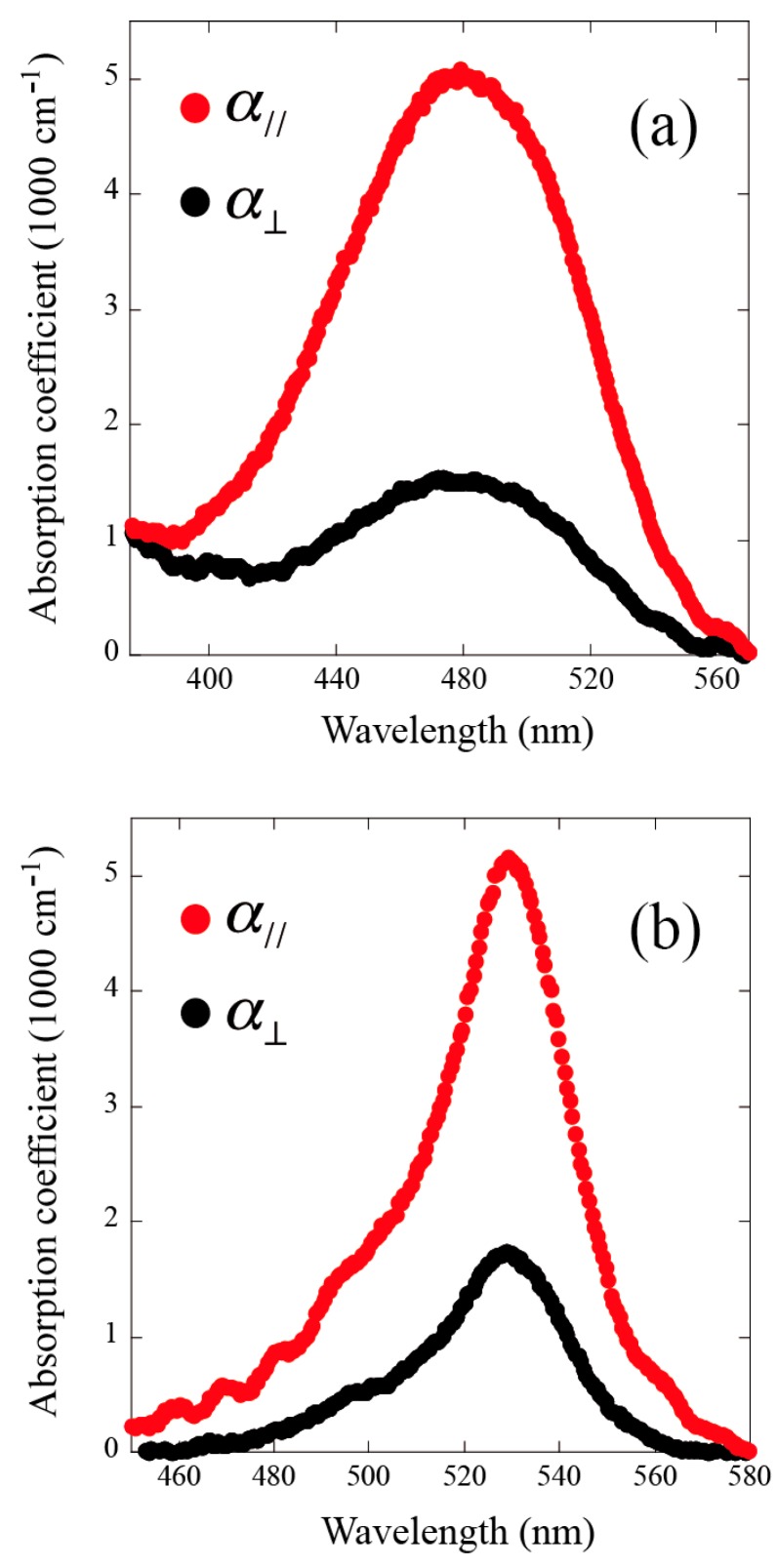
Spectra of the principal components of the absorption coefficient of an aligned nematic sample of E7 doped with DCM (1 wt %) (**a**); and with PM597 (1 wt %) (**b**).

**Figure A2:**
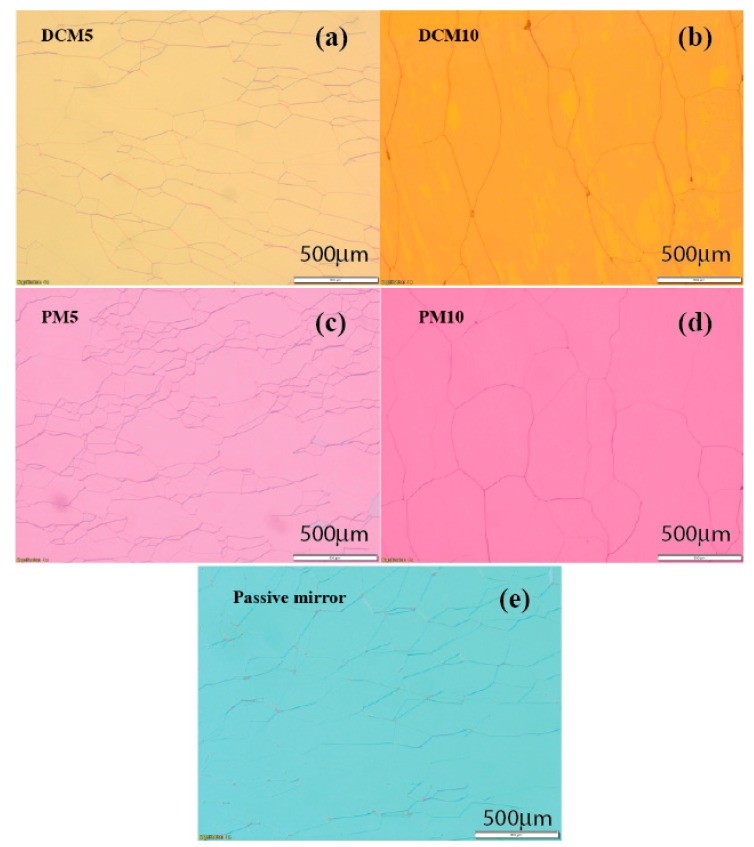
Photomicrographs of the different CLC samples used to build the complex cell architectures (**a**–**e**). The images have been obtained under crossed polarizers.

## Appendix A. Measurement of the Absorption Coefficients, Emission Cross-Sections and Scattering Losses of the Samples

The absorption coefficients of the dyes in a nematic environment were determined through transmittance measurements of linearly polarized light. Mixtures of E7 and DCM, and E7 and PM597, both in proportions 99:1 (wt %) were prepared. A fiber-based spectrometer (Avantes) was used for the measurements. The materials were enclosed in glass cells of 5 μm of thickness, whose surfaces had been rubbed in antiparallel directions. The parallel and perpendicular components of the absorption coefficients, $\alpha_{\left|\right|}$ and $\alpha_{⊥}$, were measured by illuminating the samples with linearly polarized light with the polarization direction along and perpendicular to the rubbing axis. For DCM the maxima of $\alpha_{\left|\right|}$ and $\alpha_{⊥}$ were found at *λ* = 480 nm and the anisotropy was $\alpha_{\left|\right|}/\alpha_{⊥}=3.5$, practically independent of *λ* (see Figure A1a). For PM597 the maxima were attained at 530 nm and $\alpha_{\left|\right|}/\alpha_{⊥}=3.0$ (Figure A1b). From these ratios we deduce the order parameters of the dyes in the nematic solvent from the expression:

(A1) $$S_{dye}=\frac{\alpha_{\left|\right|}/\alpha_{⊥}-1}{\alpha_{\left|\right|}/\alpha_{⊥}+2}$$

We found *S_dye_* = 0.45 and *S_dye_* = 0.40 for DCM and PM597 respectively.

The absorption coefficients at the pumping wavelength *λ* = 532 nm are shown in Table A1. The absorption cross-section for circularly polarized light (which is necessary for some of the calculations carried out in the main text) was obtained from $\sigma_{a}^{CL}=\frac{\alpha_{\left|\right|}+\alpha_{⊥}}{2N}$, where *N* is the density of dye molecules. Another interesting parameter, which is also used in the simulations, is the emission cross-section of the dye molecules along the nematic director $\sigma_{e}^{\left|\right|}$. This was obtained from the emission cross-section in the isotropic phase $\sigma_{e}^{iso}$ = 0.8 × 10^−16^ cm^2^ for both materials [52,59], using the formula:

(A2) $$\sigma_{e}^{iso}=\frac{\sigma_{e}^{\left|\right|}+2\sigma_{e}^{⊥}}{3}$$

Taking the ratio $\sigma_{e}^{\left|\right|}/\sigma_{e}^{⊥}$ equal to $\alpha_{\left|\right|}$/$\alpha_{⊥}$ and using (A1) we have

(A3) $$\sigma_{e}^{\left|\right|}=\left(1+2S_{dye}\right)\sigma_{e}^{iso}$$

The last column of Table A1 contains the values of this parameter.

**Table A1:** Absorption parameters at *λ* = 532 nm and emission cross-sections at the corresponding lasing wavelengths (605 nm for DCM and 590 nm for PM compounds).

Material	$\alpha_{\left|\right|}$ (cm^−1^)	$\alpha_{⊥}$ (cm^−1^)	$\sigma_{a}^{CL}$ (cm^2^)	$\sigma_{e}^{\left|\right|}$ (cm^2^)
E7-DCM (1%)	1736	500	0.62 × 10^−16^	1.53 × 10^−16^
E7-PM597 (1%)	5130	1700	2.3 × 10^−16^	1.44 × 10^−16^

The scattering losses of the CLC samples were determined by comparing their transmittances with those from similar cells filled with a microscope immersion oil (Cargille) of refractive index similar to that of glass. The light source was a He-Ne laser operating at 633 nm and no focusing optics was used. The light polarization was circular with opposite handedness to that of the samples in all cases. At this wavelength the CLC samples do not show appreciable absorption, and all intensity losses should be due to scattering. The transmitted signals were measured with a detector placed at about 1 m from the samples, thus assuring a small angular aperture. The data were analyzed assuming a law of the type *I* = *I*_0_ exp(−*βL*) for the light intensity *I*, where *I*_0_ is the intensity transmitted by the immersion-oil sample. Here *L* is the CLC cell thickness and *β* the scattering coefficient.

We studied 5 different samples, and in all of them we found some irregular behavior in the transmission values as their surfaces were scanned. This effect is likely to be originated by local defects in the sample alignments. This idea is in accordance with the correlation found between the amount of defects observed in the studied samples and the corresponding *β* values (see Figure A2). Thus, data in Table A2 must be considered just as approximate averages. The intrinsic scattering coefficient of a perfect sample is probably smaller.

**Table A2:** Scattering parameters at *λ* = 633 nm.

Sample	DCM10	PM10	Passive Mirror 5 μm	DCM5	PM5
*β* (cm^−1^)	150	150	300	300	300

## References

[1] Kopp V.I., Fan B., Vithana H.K.M., Genack A.Z. (1998). Low-threshold lasing at the edge of a photonic stop band in cholesteric liquid crystals. Opt. Lett..

[2] Kopp V.I., Zhang Z.Q., Genack A.Z. (2003). Lasing in chiral photonic structures. Prog. Quantum Electron..

[3] Blinov L.M., Bartolino R. (2010). Liquid Crystal Microlasers.

[4] Takezoe H., Li Q. (2012). Liquid crystal lasers. Liquid Crystals beyond Displays.

[5] Coles H., Morris S. (2010). Liquid-crystal lasers. Nat. Photonics.

[6] Schmidtke J., Stille W. (2003). Fluorescence of a dye-doped cholesteric liquid crystal film in the region of the stop band: Theory and experiment. Eur. Phys. J. B.

[7] Beeckman J., Neyts K., Vanbrabant P.J. (2011). Liquid-crystal photonic applications. Opt. Eng..

[8] Cao W., Palffy-Muhoray P., Taheri B., Marino A., Abbate G. (2005). Lasing thresholds of cholesteric liquid crystals lasers. Mol. Cryst. Liq. Cryst..

[9] Morris S.M., Ford A.D., Gillespie C., Pivnenko M.N., Hadeler O., Coles H.J. (2006). The emission characteristics of liquid-crystal lasers. J. SID.

[10] Sanz-Enguita G., Ortega J., Folcia C.L., Aramburu I., Etxebarria J. (2016). Role of the sample thickness on the performance of cholesteric liquid crystal lasers: Experimental, numerical, and analytical results. J. Appl. Phys..

[11] Coles H.J., Morris S.M., Ford A.D., Hands P.J.W., Wilkinson T.D. Red-Green-Blue 2D Tuneable Liquid Crystal Laser Devices. Proceedings of the SPIE Photonic Devices + Applications.

[12] Etxebarria J., Ortega J., Folcia C.L., Sanz-Enguita G., Aramburu I. (2015). Thermally induced light-scattering effects as responsible for the degradation of cholesteric liquid crystal lasers. Opt. Lett..

[13] Schmidtke J., Stille W., Finkelmann H., Kim S.T. (2002). Laser emission in a dye doped cholesteric polymer network. Adv. Mater..

[14] Morris S.M., Qasim M.M., Gardiner D.J., Hands P.J.W., Castles F., Tu G., Huck W.T.S., Friend R.H., Coles H.J. (2013). Liquid crystalline chromophores for photonic band-edge laser devices. Opt. Mater..

[15] Mowatt C., Morris S.M., Song M.H., Wilkinson T.D., Friend R.H., Coles H.J. (2010). Comparison of the performance of photonic band-edge liquid crystal lasers using different dyes as the gain medium. J. Appl. Phys..

[16] Ford A.D., Morris S.M., Pivnenko M.N., Gillespie C., Coles H.J. (2007). Emission characteristics of a homologous series of bimesogenic liquid-crystal lasers. Phys. Rev. E.

[17] Vasnetsov M.V., Slussarenko S.S., Stumpe J., Sakhno O., Slussarenko S.S., Abbate G. (2010). Lasing by Second-Order Bragg Diffraction in Dye-Doped POLIPHEM Gratings. Mol. Cryst. Liq. Cryst..

[18] Qi J., Crawford G.P. (2004). Holographically formed polymer dispersed liquid crystal displays. Displays.

[19] Abbate G., Vita F., Marino A., Tkachenko V., Slussarenko S., Sakhno O., Stumpe J. (2006). New generation of holographic gratings based on polymer-LC composites: POLICRYPS and POLIPHEM. Mol. Cryst. Liq. Cryst..

[20] Ko D.H., Morris S.M., Lorenz A., Castles F., Butt H., Gardiner D.J., Qasim M.M., Wallikewitz B., Hands P.J.W., Wilkinson T.D. (2013). A nano-patterned photonic crystal laser with a dye-doped liquid crystal. Appl. Phys. Lett..

[21] Morris S.M., Gardiner D.J., Hands P.J.W., Qasim M.M., Wilkinson T.D., White I.H., Coles H.J. (2012). Electrically switchable random to photonic band-edge laser emission in chiral nematic liquid crystals. Appl. Phys. Lett..

[22] Li L., Deng L. (2013). Low threshold and coherent random lasing from dye-doped cholesteric liquid crystals using oriented cells. Laser Phys..

[23] Strangi G., Barna V., De Luca A., Ferjani S., Versace C., Blinov L.M., Bartolino R. (2010). Random lasing in liquid crystals. Liquid Crystal Microlasers.

[24] Penninck L., Beeckman J., De Visschere P., Neyts K. (2012). Light emission from dye-doped cholesteric liquid crystals at oblique angles: Simulation and experiment. Phys. Rev. E.

[25] Morris S.M., Hands P.J.W., Findeisen-Tandel S., Cole R.H., Wilkinson T.D., Coles H.J. (2008). Polychromatic liquid crystal laser arrays towards display applications. Opt. Express.

[26] Barberi R., Chilaya G., Blinov L.M., Bartolino R. (2010). Strategies for tunable cholesteric lasers. Liquid Crystal Microlasers.

[27] Yu H., Tang B.Y., Li J., Li L. (2005). Electrically tunable lasers made from electro-optically active photonics band gap materials. Opt. Express.

[28] Park B., Kim M., Kim S.W., Jang W., Takezoe H., Kim Y., Choi E.H., Seo Y.H., Cho G.S., Kang S.O. (2009). Electrically Controllable Omnidirectional Laser Emission from a Helical-Polymer Network Composite Film. Adv. Mater..

[29] Schmidtke J., Jünnemann G., Keuker-Baumann S., Kitzerow H.S. (2012). Electrical fine tuning of liquid crystal lasers. Appl. Phys. Lett..

[30] Xiang J., Varanytsia A., Minkowski F., Paterson D.A., Storey J.M.D., Imrie C.T., Lavrentovich O.D., Palffy-Muhoray P. (2016). Electrically tunable laser based on oblique heliconical cholesteric liquid crystal. Proc. Natl. Acad. Sci. USA.

[31] Chen L.J., Lin J.D., Lee C.R. (2014). An optically stable and tunable quantum dot nanocrystal-embedded cholesteric liquid crystal composite laser. J. Mater. Chem. C.

[32] Mykytiuk T.V., Ilchishin I.P., Yaroshchuk O.V., Kravchuk R.M., Li Y., Li Q. (2014). Rapid reversible phototuning of lasing frequency in dye-doped cholesteric liquid crystal. Opt. Lett..

[33] Chilaya G.S. (2006). Light-controlled change in the helical pitch and broadband tunable cholesteric liquid-crystal lasers. Crystallogr. Rep..

[34] Finkelmann H., Kim S.T., Muñoz A., Palffy-Muhoray P., Taheri B. (2001). Tunable mirrorless lasing in cholesteric liquid crystalline elastomers. Adv. Mater..

[35] Huang Y., Zhou Y., Doyle C., Wu S.T. (2006). Tuning the photonic band gap in cholesteric liquid crystals by temperature-dependent dopant solubility. Opt. Express.

[36] Funamoto K., Ozaki M., Yoshino K. (2003). Discontinuous shift of lasing wavelength with temperature in cholesteric liquid crystal. Jpn. J. Appl. Phys..

[37] Oldano C., Reyes J.A., Ponti S. (2002). Twist defects in helical sonic structures. Phys. Rev. E.

[38] Kopp V.I., Genack A.Z. (2002). Twist Defect in Chiral Photonic Structures. Phys. Rev. Lett..

[39] Gevorgyan A.H. (2009). Specific features of the emission of chiral photonic crystals with an anisotropic defect: I. Thickness effects. Opt. Spectrosc..

[40] Belyakov V.A., Semenov S.V. (2011). Optical defect modes in chiral liquid crystals. J. Exp. Theor. Phys..

[41] Matsui T., Kitaguchi M. (2012). Finite-Difference Time-Domain Analysis of Twist-Defect-Mode Lasing Dynamics in Cholesteric Photonic Liquid Crystal. Jpn. J. Appl. Phys..

[42] Schmidtke J., Stille W., Finkelmann H. (2003). Defect mode emission of a dye doped cholesteric polymer network. Phys. Rev. Lett..

[43] Jeong S.M., Ha N.Y., Takanishi Y., Ishikawa K., Takezoe H., Nishimura S., Suzaki G. (2007). Defect mode lasing from a double-layered dye-doped polymeric cholesteric liquid crystal films with a thin rubbed defect layer. Appl. Phys. Lett..

[44] Yoshida H., Lee C.H., Matsuhisa Y., Fujii A., Ozaki M. (2007). Bottom-Up fabrication of photonic defect structures in cholesteric liquid crystals based on laser-assisted modification of the helix. Adv. Mater..

[45] Song M.H., Park B.C., Shin K.C., Ohta T., Tsunoda Y., Hoshi H., Takanishi Y., Ishikawa K., Watanabe J., Nishimura S. (2004). Effect of phase retardation on defect-mode lasing in polymeric cholesteric liquid crystals. Adv. Mater..

[46] Zhou Y., Jang E.A., Huang Y., Wu S. (2007). Enhanced laser emission in opposite handedness using a cholesteric polymer film stack. Opt. Express.

[47] Takanishi Y., Tomoe N., Ha N.Y., Toyooka T., Nishimura S., Ishikawa K., Takezoe H. (2007). Defect-Mode Lasing from a Three-Layered Helical Cholesteric Liquid Crystal Structure. Jpn. J. Appl. Phys..

[48] Song M.H., Ha N.Y., Amemiya K., Park B., Takanishi Y., Ishikawa K., Wu J.W., Nishimura S., Toyooka T., Takezoe H. (2006). Defect-mode lasing with lowered threshold in a three-layered hetero-cholesteric liquid-crystal structure. Adv. Mater..

[49] Zhou Y., Huang Y., Ge Z., Chen L., Hong Q., Wu T.X., Wu S. (2006). Enhanced photonic band edge laser emission in a cholesteric liquid crystal resonator. Phys. Rev. E.

[50] Muñoz A., McConney M.E., Kosa T., Luchette P., Sukhomlinova L., White T.J., Bunning T.J., Taheri B. (2012). Continuous wave mirrorless lasing in cholesteric liquid crystals with a pitch gradient across the cell gap. Opt. Lett..

[51] Morris S.M., Ford A.D., Pivnenko M.N., Hadeler O., Coles H.J. (2006). Correlations between the performance characteristics of a liquid crystal laser and the macroscopic material properties. Phys. Rev. E.

[52] Blinov L.M. (2009). Lasers on cholesteric liquid crystals: Mode density and lasing threshold. JETP Lett..

[53] Penninck L., Beeckman J., De Visschere P., Neyts K. (2013). Numerical simulation of stimulated emission and lasing in dye doped cholesteric liquid crystal films. J. Appl. Phys..

[54] Shtykov N.M., Palto S.P. (2014). Modeling laser generation in cholesteric liquid crystals using kinetic equations. JETP.

[55] Berreman D.W. (1972). Optics in stratified and anisotropic media: 4 × 4-Matrix formulation. J. Opt. Soc. Am..

[56] Belyakov V.A., Semenov S.V. (2009). Optical edge modes in photonic liquid crystals. JETP.

[57] Shtykov N.M., Palto S.P., Umanskii B.A. (2013). Simulation of light generation in cholesteric liquid crystals using kinetic equations: Time-independent solution. JETP.

[58] Lub J., Nijssen W.P.M., Wegh R.T., De Francisco I., Ezquerro M.P., Malo B. (2005). Photoisomerizable chiral compounds derived from isosorbide and cinnamic acid. Liq. Cryst..

[59] Bañuelos Prieto J., López Arbeloa F., Martínez Martínez V., Arbeloa López T., López Arbeloa I. (2004). Photophysical properties of the pyrromethene 597 dye: Solvent effect. J. Phys. Chem. A.

[60] Montejano H.A., Amat-Guerri F., Costela A., García-Moreno I., Liras M., Sastre R. (2006). Triplet-state spectroscopy of dipyrromethene·BF_2_ laser dyes. J. Photochem. Photobiol. A Chem..

